# Prevalence, awareness, and associated factors of high blood pressure among female migrant workers in Central South China

**DOI:** 10.7717/peerj.13365

**Published:** 2022-05-04

**Authors:** Hua Peng, Mei Sun, Xin Hu, Huiwu Han, Jing Su, Emin Peng, James Wiley, Lisa Lommel, Jyu-Lin Chen

**Affiliations:** 1 Department of Nursing, Xiangya Hospital of Central South University, Changsha, China; 2 Community Nursing Department, Xiangya Nursing School of Central South University, Hunan Women Research Association, Changsha, China; 3 Community Nursing Department, Xiangya Nursing School of Central South University, Changsha, China; 4 Department of Cardiology, Xiangya Hospital of Central South University, Changsha, China; 5 Department of Nursing, Shantou University Medical College, Shantou, China; 6 Outpatient Clinic, Xiangya Hospital of Central South University, Changsha, China; 7 Institute for Health Policy Studies, University of California, San Francisco, CA, United States of America; 8 Department of Family Health Care Nursing, School of Nursing, University of California, San Francisco, CA, United States of America

**Keywords:** Hypertension, Migrant workers, Prevalence, Awareness

## Abstract

**Background:**

Although many young Chinese women migrate to urban regions for better opportunities, little is known about the prevalence and awareness of having high blood pressure (HBP) in this population. This study investigated the prevalence, awareness, and factors associated with HBP among young female migrant workers in Central South China.

**Methods:**

We conducted a cross-sectional study to identify HBP (2017 ACC/AHA guidelines) among female migrant workers aged 18–45 years in Central South China. Demographics, anthropometric measurements, hypertension-related lifestyle, awareness of HBP, and blood pressure were recorded. Logistic regression analysis was used to identify the factors associated with HBP (blood pressure ≥ 130/80 mmHg).

**Results:**

Overall, 232 female migrants participated in the study (mean age 34.4; standard deviation: 6.4 years). The prevalence of HBP was 27.2% (95% CI [21.6–33.2]), and 88.9% of the participants were unaware of their HBP status. Having rural medical insurance (odds ratio [OR] = 20.7; 95% confidence interval 95% CI [2.1–204.8]), awareness of having HBP (OR = 5.1; 95% CI [1.4–18.5]), physical inactivity (OR = 2.9; 95% CI [1.1–7.9]), and being overweight/obese (OR = 2.7; 95% CI [1.3–6.1]) were independently associated with HBP.

**Conclusions:**

This study revealed a high prevalence of HBP among young Chinese female migrant workers, as well as a high frequency of being unaware of their condition and some associated factors (rural medical insurance, awareness of having HBP, physical inactivity, and overweight/obesity). The uncontrolled HBP among young Chinese female migrant workers suggested that health education needs further promotion in such a population.

## Introduction

Hypertension is a leading cause of cardiovascular diseases (CVD) worldwide (Roth et al., 2020; Wu et al., 2015) and is also strongly associated with many cerebrovascular diseases such as stroke (Roth et al., 2020; Wajngarten & Silva, 2019). The Chinese hypertension guidelines are similar to the recommendations from the Eighth Joint National Committee (JNC-8) (James et al., 2014; Wang, 2015), defining hypertension as systolic blood pressure (SBP) ≥ 140 mmHg or diastolic blood pressure (DBP) ≥ 90 mmHg, or use of antihypertensive medication, while high blood pressure (HBP) is defined as blood pressure that is high but without necessarily qualifying for hypertension, *i.e.*, SBP ≥ 130 mmHg or DBP ≥ 80 mmHg (Li et al., 2019). The high prevalence of hypertension significantly aggravates the public health burden demonstrated in morbidity, mortality, and medical care expenses, particularly in low- and middle-income countries, including China (Sarki et al., 2015; van Kleef & Spiering, 2017). As well as having the largest proportion of the world’s population, China has experienced a rapidly increased prevalence of hypertension among adults aged ≥ 18 years, from 26.6% in 1990 to 33.6% in 2016 (Bundy & He, 2016). According to the most recent national survey on adults aged 35–75 years in China (in 2017), the prevalence of hypertension has reached 44.7%, but the overall rates for awareness of having HBP, hypertension treatment, and control (<140/90 mmHg) were poor (44.7%, 30.1%, and 5.7%, respectively) (Lu et al., 2017).

As prevention of hypertension is key to preventing CVD, the clinical practice guidelines for detecting and treating hypertension were updated in 2017 by the American College of Cardiology/American Heart Association (ACC/AHA) (Whelton et al., 2018). By lowering the blood pressure level for hypertension diagnosis, the new 2017 guidelines targeted BP reduction in adults at high risk of hypertension or adults with hypertension to prevent developing HBP-related CVD, especially adults who are overweight, consume excessive dietary sodium and alcohol, or those who are physically inactive (Whelton et al., 2018). Considering the importance of early prevention for hypertension management of young patients, this study used the new definition of hypertension stage I recommended by the ACC/AHA as the cutoff point (SBP ≥ 130 mmHg or DBP ≥ 80 mmHg) for HBP.

Mainland China has the largest population of native migrant workers (*i.e.*, born in China and migrated to another region/province for work) globally, with most of them being rural-to-urban migrants (National Bureau of Statistics of the People’s Republic of China, 2016). In 2016, rural-to-urban migrant workers had increased to 281.7 million, accounting for 20.4% of the total Chinese population (National Bureau of Statistics of the People’s Republic of China, 2017). Young migrants aged 26–40 years having a greater prevalence of prehypertension (SBP 120–139 mmHg and/or DBP 80–89 mmHg) compared to older migrants (≥ 41 years) has been observed (Guan, 2018). Ying et al. (2015) reported that young migrant people aged ≥ 30 years had a higher prevalence of hypertension than migrant adults aged <30 years (12.4% *vs.* 5.5%, *p* = 0.001; odds ratio [OR]: 2.0, 95% CI [1.3–3.0]). Many studies have found a higher prevalence of hypertension in people aged ≥ 35 years than in those <35 years (Donghui et al., 2015; Gao et al., 2013; Hu, Gao & Liu, 2019; Li et al., 2019; Liu et al., 2017). Still, it remains unclear whether the epidemiology and risk factors of HBP among women migrant workers are the same as in the general population.

Lifestyle modification could significantly reduce the prevalence of HBP and hypertension, especially in urban dwellers. The potentially modifiable factors related to lifestyle include smoking cessation, restricted alcohol intake, increased fruit and vegetable consumption, weight loss, increased physical activity, and reduced dietary salt intake (Juraschek et al., 2017; Masterson Creber et al., 2010; Zhou, 2002; Zhou et al., 2002). Bi et al. (2016) suggested that obesity might be the earliest manifestation of hypertension and CVD among Chinese migrant workers after transitioning to an urbanized lifestyle. Another study found that long working hours (>8 hours/day), higher income (>USD 490/month), and single marital status are associated with unhealthy lifestyles in female migrant workers (Yang et al., 2015a). Understanding the prevalence of hypertension and health behaviors related to hypertension, which is the leading cause of CVD, is critical in eliminating health disparities in this young and understudied population.

Therefore, this study aimed (a) to estimate the prevalence and awareness of having HBP and (b) to identify factors associated with HBP among female migrant workers from Central South China.

## Method

### Study design and setting

This cross-sectional study was conducted in Changsha, Hunan Province, Central South China, between March 2017 and June 2017. Hunan has a total population of 68.99 million, with 38.65 million (56.0%) urban and 30.34 million (44.0%) rural residents by the end of 2018. However, the number of domestic migrant workers in Hunan has reached approximately 17.58 million, accounting for approximately 25.5% of the total provincial population (Hunan Provincial Bureau of Statistics, 2018). Hunan ranked third in the total number of migrant workers in China and first in Central South China (National Bureau of Statistics of China, 2020). Among the migrant population in Hunan, nearly 9.59 million (54.6% of the total migrants in Hunan) were relatively young, born after 1980. Changsha is the biggest commercial, transportation, and manufacturing center in Hunan, with approximately 8.15 million people in 2018 (Hunan Provincial Bureau of Statistics, 2018).

According to the Chinese National Bureau of Statistics, all qualified workplaces were classified into three levels: large, ≥ 1,000 employees; medium, 300–999 employees; and small, 10–299 employees (National Bureau of Statistics of China, 2020). Workplaces with female migrant workers accounting for 50% of the total employees or above were eligible.

The institutional review board of the University of California, San Francisco, and Central South University reviewed and approved the study protocol (ID: 2017036). Patients’ personal information and anthropometric data are kept strictly confidential and only used for scientific research. The participants all signed the informed consent form.

### Study population

Mandarin-speaking research assistants recruited a convenience sample of young female participants from custodians in a tertiary hospital, workers in a medical device factory, and staff in a restaurant in Changsha, representing large-, medium-, and small-sized workplaces, respectively, considering that these migrant women are mainly engaged in the labor-intensive light industry and service sectors due to their traits of physical strength and education. The inclusion criteria were (a) Chinese female migrants from rural regions working in Changsha, (b) age between 18 and 45 years, (c) able to read and write in Mandarin Chinese, and (d) has stayed in Changsha as a migrant worker for at least 6 months. Participants were not recruited if they could not provide consent due to physical or mental illness or declined to participate.

### Sample size

The sample size was estimated using an online sample size calculator for prevalence studies (https://www.calculator.net/sample-size-calculator.html). Assuming that the prevalence of hypertension in the female migrant workers was similar to the reported prevalence of 15.6% (Wang et al., 2018) in the population aged ≥ 18 years in Hunan province in 2012–2015, we calculated the minimum sample size by taking 5% permission error with 95% confidence interval (CI) and the population size as 8.15 million. Considering the nonresponse rate of 20%, a final sample size of 254 was calculated.

### Measurements

All participants were informed about the purpose of the study and details of their enrollment by trained interviewers, and they provided written informed consent at their workplace. After completing the screening questionnaire, each subject’s weight, height, waist circumference (WC), and blood pressure were measured by trained research assistants in a separate room to ensure privacy. All measurements and screenings were completed at the place of their employment. The interviewers were trained by the research principal investigator to perform the procedure in a standardized manner, collect data, and interpret the questionnaire in standard language. Meanwhile, the research assistants were trained about physical measurements by a professional cardiac clinical instructor. Each participant received a small umbrella as a token of appreciation for their participation.

### Demographic survey

A questionnaire was developed to examine the sociodemographic characteristics of the sample population, including their age (≥35 or <35 years), the highest level of school (higher than high school *vs.* high school or less), marital status (married, single, or widowed), family location (village or town), whether a mother with children (yes or no), job category (manager or worker), the length of being a migrant worker (<1, 1–3, 4–5, or >5 years), average monthly income (≤420 or >420 USD), and hours worked per day (≤8 or >8 h).

The participants were also asked about whether they had rural medical insurance (yes or no), had ever taken HBP pills (yes or no), and received an annual health examination (yes or no). Regarding whether their parents had ever been diagnosed with CVD, the responses included yes, no, or don’t know.

### Survey on hypertension-related lifestyle

The questionnaire also obtained lifestyle behaviors, including dietary behaviors, physical activity level, and cigarette smoking status. Items related to dietary behaviors included whether the participants consumed five servings of fruits and vegetables per day, were told by a physician or a nurse to reduce salt intake, or were told by a physician or a nurse to reduce carbohydrate intake. Physical activity level was assessed by asking participants how many days per week they engage in regular physical exercise of ≥30 min per session. The responses included ≥3 days per week and <3 days per week. Regarding smoking status, information was collected about whether they were current smokers (yes or no). These lifestyle behavior questions are included in the Chinese version of the Canadian Diabetes Risk Questionnaire, which has been confirmed to have good reliability and validity (Guo et al., 2018).

### Awareness of having HBP and self-rated health

The question assessed the awareness of having HBP, “Have you ever been told by a physician or nurse that your blood pressure was high?” Overall health was self-rated by participants as poor, fair, good, very good, or excellent.

### Blood pressure

Blood pressure was measured using an OMRON automatic blood pressure monitor (HBP-1100; Omron Corporation, Dalian, China) and validated using mercury sphygmomanometer measurements. The participants had to avoid caffeine, exercise, and smoking for at least 30 min before their visit. They were asked to empty their bladder before entering the test room. The participants were told to relax and rest in the seated position (feet on the floor and back supported) for 10 min, without talking, and two blood pressure measurements were obtained at 60-s intervals, also without talking. Blood pressure measurements were made on the left arm, with the transducer placed at the level of the heart and the cuff adapted to the arm circumference. The final blood pressure result was the arithmetic mean of the two measurements taken on the same arm. The precision of the automatic blood pressure monitor was one mmHg. The participants were considered to have HBP if the SBP was ≥130 mmHg or the DBP was ≥80 mmHg (including stage I and above). Hypertension was defined as having HBP or self-reported antihypertensive medication use.

### Body composition

Body composition was assessed by measuring the body mass index (BMI). The weight (kg) and height (cm) of the participants were measured, and BMI was calculated as weight in kilograms divided by the square of height in meters (weight/height^2^ (kg/m^2^)). With the participants wearing light clothing and no shoes, weight was measured to the nearest 0.5 kg in increments of up to 120 kg using the Penguin TCS weighing scale (TCS-766; Wuyi Penguin Industry and Trade Co. Ltd., Zhejiang, China). Height was measured to the nearest 0.5 cm using a stadiometer, with the participant not wearing footwear. Overweight (24 kg/m^2^ ≤ BMI <28 kg/m^2^) and obesity (BMI ≥ 28 kg/m^2^) were identified using standard Chinese-specific BMI cutoff values (Jin et al., 2013; Yang et al., 2017).

### Waist circumference

The WC was measured using standard protocols. With the participant in a standing position, the abdomen was fully exposed, and measurement was conducted in a state of natural respiration. The WC was determined as the smallest measure of the waist at the level of the umbilicus by using a nonelastic plastic ribbon with a width of 0.7 cm. The reading was measured to the nearest 0.1 cm. Based on previous studies, the participants were defined to have central obesity when the WC was ≥80 cm (Du et al., 2017; Xu et al., 2016).

### Statistical analysis

The participants’ sociodemographic characteristics were presented using descriptive statistics, including frequencies, percentages, and means with standard deviations (SDs). Bivariable logistic regression analyses were performed to examine the odds ratio (OR) and 95% CI of hypertension by separate parameters. A multivariable logistic regression analysis was performed to estimate the OR and 95% CI of hypertension after adjustment for variables such as age, education, cigarette use, physical activity, fruit and vegetable intake, receiving advice to reduce salt and carbohydrates, parental history of CVD, WC, BMI, awareness of having HBP and rural medical insurance. A directed acyclic graph is a method to illustrate causal relationships between variables based on professional and epidemiological knowledge (Etminan, Collins & Mansournia, 2020). Then, the variance inflation factor (VIF) was used to check the collinearity of the selected variables. VIF <5 indicated that collinearity was unlikely to be a problem (Gueugnon et al., 2019; Kim, 2019; Marcoulides & Raykov, 2019). The multivariable regression model for the outcome (HBP) was described using a receiver operating characteristic (ROC) curve and the area under the curve (AUC) (Hajian-Tilaki, 2013; Zou, O’Malley & Mauri, 2007). The Hosmer-Lemeshow test and calibration curves were used to test the calibration of the model. All *P*-values were two-sided and considered statistically significant at <0.05. Statistical analyses were conducted using IBM SPSS Statistics v.25.0 (International Business Machines Corp., Armonk, New York, USA).

## Results

### Participant characteristics

**Figure 1:**
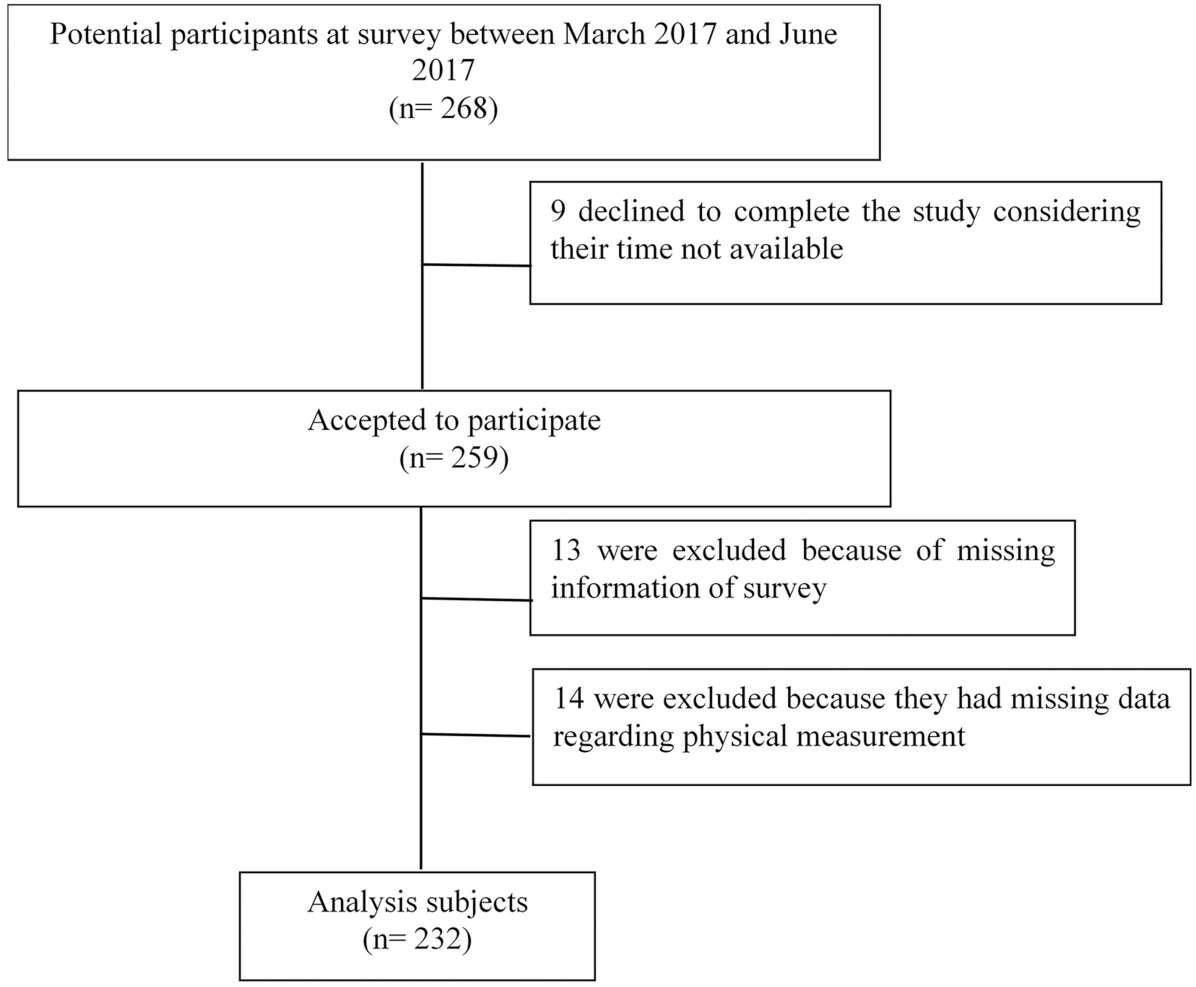
Flowchart.

Of the 268 participants considered for participation, nine declined to participate in the study because of time constraints, thirteen did not complete the questionnaire, and fourteen missed at least one of the physical measurements. The overall response rate was 86.6% (232 participants), with missing data in <5% of the participants (Fig. 1). There were 167 custodians from a tertiary hospital, 51 workers from a medical device factory, and 14 staff in a restaurant in Changsha, representing large-, medium-, and small-sized workplaces, respectively.

Of the 232 female migrant workers who participated in this study, 157 (67.7%) migrated from rural villages, while 75 (32.3%) migrated from small towns located in suburban areas where the living conditions are more similar to rural conditions. The mean age of the participants was 34.4 years (SD = 6.4, range 18–45 years). Most of the migrant women had a high school diploma or less education (88.8%), were married and also had children (79.7%), and made <USD 420/month (77.2%). Additionally, about one-third of these women reported working >8 hours/day, and one-half has migrated for more than 5 years (Table 1).

**Table 1:** Characteristics of the participants, frequencies (percentage) [n (%)].

Characteristic	Total (*n* = 232)	High blood pressure^a^	
		No (*n* = 169)	Yes (*n* = 63)
Age (years)			
<35	127 (54.7)	100 (59.2)	27 (42.9)
≥35	105 (45.3)	69 (40.8)	36 (57.1)
Education			
Higher than high school	26 (11.2)	21 (12.4)	5 (7.9)
High school or less	206 (88.8)	148 (87.6)	58 (92.1)
Marital status			
Married	212 (91.4)	153 (90.5)	59 (93.7)
Single/widowed	20 (8.6)	16 (9.5)	4 (6.3)
Family location			
Village	157 (67.7)	114 (67.5)	43 (68.3)
Town	75 (32.3)	55 (32.5)	20 (31.7)
Mother with children			
No	47 (20.3)	33 (19.5)	14 (22.2)
Yes	185 (79.7)	136 (80.5)	49 (77.8)
Job category			
Manager	33 (14.2)	26 (15.4)	7 (11.1)
Worker	199 (85.8)	143 (84.6)	56 (88.9)
Length of migration (years)			
<1	25 (10.8)	17 (10.1)	8 (12.7)
1–3	59 (25.4)	47 (27.8)	12 (19.0)
4–5	21 (9.1)	13 (7.7)	8 (12.7)
>5	127 (54.7)	92 (54.4)	35 (55.6)
Monthly income			
≤420 USD	179 (77.2)	131 (77.5)	48 (76.2)
>420 USD	53 (22.8)	38 (22.5)	15 (23.8)
Hours worked per day			
≤8	147 (63.4)	111 (65.7)	36 (57.1)
>8	85 (36.6)	58 (34.3)	27 (42.9)
Rural medical insurance			
No	33 (14.2)	32 (18.9)	1 (1.6)
Yes	199 (85.8)	137 (81.1)	62 (98.4)
Annual health examination			
No	95 (40.9)	74 (43.8)	21 (33.3)
Yes	137 (59.1)	95 (56.2)	42 (66.7)
Parents with CVD			
No/do not know	196 (84.5)	142 (84.0)	54 (85.7)
Yes	36 (15.5)	27 (16.0)	9 (14.3)
Consume 5 servings of fruits/vegetables per day			
No	149 (64.2)	108 (63.9)	41 (65.1)
Yes	83 (35.8)	61 (36.1)	22 (34.9)
Advised to reduce salt intake			
No	152 (65.5)	114 (67.5)	38 (60.3)
Yes	80 (34.5)	55 (32.5)	25 (39.7)
Advised to reduce carbohydrate intake			
No	189 (81.5)	139 (82.2)	50 (79.4)
Yes	43 (18.5)	30 (17.8)	13 (20.6)
Physical activity ≥ 3 days/week			
No	189 (81.5)	132 (78.1)	57 (90.5)
Yes	43 (18.5)	37 (21.9)	6 (9.5)
Current smoker			
No	229 (98.7)	168 (99.4)	61 (96.8)
Yes	3 (1.3)	1 (0.6)	2 (3.2)
Self-rated overall health			
Good/ very good/ excellent	135 (58.2)	103 (60.9)	32 (50.8)
Fair/poor	97 (41.8)	66 (39.1)	31 (49.2)
Body mass index (kg/m^2^)			
<24	169 (72.8)	132 (78.1)	37 (58.7)
≥24	63 (27.2)	37 (21.9)	26 (41.3)
Waist circumference (cm)			
<80	129 (55.6)	103 (60.9)	26 (41.3)
≥80	103 (44.4)	66 (39.1)	37 (58.7)

**Notes.**

a SBP ≥ 130 mmHg or DBP ≥ 80 mmHg.

CVD cardiovascular disease DBP diastolic blood pressure SBP systolic blood pressure USD United States dollar

The mean BMI was 22.17 kg/m^2^ (SD = 3.28), with 27.2% of the migrant women being overweight (22.4%) or obese (4.7%). The mean WC was 79.30 cm (SD = 8.20), with 44.4% of the participants meeting the cutoff for central obesity (≥80 cm).

### Prevalence/awareness of having HBP and lifestyle behaviors

The prevalence of HBP and hypertension (with HBP or antihypertensive medication use) was 27.2% (63/232; 95% CI [21.6–33.2]) and 28.9% (67/232; 95% CI [23.3–34.9]), respectively. Of the female migrant workers with HBP, 69.8% (44/63) had stage I hypertension, while 30.2% (19/63) had stage II hypertension (Table 2).

**Table 2:** Distribution of blood pressure among the female migrant workers in Central South China, frequencies (percentage) [n (%)].

BP category (SBP/DBP, mmHg)	n (%)	95% CI^*^
Normal (<120/80)	158 (68.1)	62.1–74.1
Elevated (120–129/<80)	11 (4.7)	2.2–7.3
Hypertension stage I (130–139 /or 80–89)	44 (19.0)	14.2–24.1
Hypertension stage II (≥ 140 /or ≥ 90)	19 (8.2)	4.7–12.5

**Notes.**

DBP diastolic blood pressure SBP systolic blood pressure CI confidence interval

a 95% CI of the percentage.

Approximately 5.6% (13/232) of female migrants believed that they currently or formerly had HBP (Table 3). Notably, 88.9% of the female migrants with HBP reported that they were unaware of their HBP status, and only four used antihypertensive medication (Table 3). Moreover, 50.8% of these workers who had HBP self-rated their overall health status as good, very good, or excellent. Overall, 59.1% had undergone annual health physical examinations, 1.3% reported smoking, 64.2% reported consuming less than five servings of fruits and vegetables daily, and 81.5% reported not engaging in physical activity at least three days per week.

**Table 3:** Awareness and treatment of participants.

	High blood pressure^a^		*P*
	No (*n* = 169)	Yes (*n* = 63)	
Awareness			0.026
Yes	6 (3.6)	7 (11.1)	
No	163 (96.4)	56 (88.9)	
Antihypertensive medication use			0.218
Yes	4 (2.4)	4 (6.3)	
No	165 (97.6)	59 (93.7)	

**Notes.**

Data displayed as frequency (percentage) [n (%)].

a SBP ≥ 130 mmHg or DBP ≥ 80 mmHg.

Additionally, among the 67 participants with hypertension (SBP ≥ 130 mmHg or DBP ≥ 80 mmHg or antihypertensive medication use), 83.6% were unaware of it, 11.9% used antihypertensives, and 94.0% had uncontrolled blood pressure.

### Factors associated with HBP

The bivariable analyses showed that age ≥ 35 years, physical activity <3 days/week, waist circumference ≥ 80 cm, BMI ≥ 24 kg/m^2^, awareness of having HBP, and having rural medical insurance were associated with higher odds of HBP (all *P* < 0.05, Table 4).

**Table 4:** Logistic regressions for high blood pressure among female migrant workers in China.

Variables	Bivariable analysis			Multivariable analysis		
	OR	95% CI	*P*	OR	95% CI	*P*
Age (years)						
<35	reference			reference		
≥35	1.93	1.08–3.47	0.028	1.33	0.66–2.69	0.426
Education						
Higher than high school	reference			reference		
High school or less	1.65	0.59–4.57	0.339	1.23	0.40–3.81	0.714
Physical activity ≥ 3 days/week						
No	2.66	1.07–6.66	0.036	2.94	1.09–7.90	0.033
Yes	reference			reference		
Consume 5 servings of fruits/vegetables per day						
No	1.05	0.57–1.93	0.868	1.32	0.66–2.62	0.437
Yes	reference			reference		
Current smoker						
No	reference			reference		
Yes	5.51	0.49–61.84	0.167	3.29	0.25–42.48	0.362
Advised to reduce salt intake						
No	reference			reference		
Yes	1.36	0.75–2.48	0.310	1.97	0.83–4.68	0.125
Advised to reduce carbohydrate intake						
No	reference			reference		
Yes	1.21	0.58–2.49	0.615	0.46	0.16–1.30	0.142
Parents with CVD						
No/do not know	1.14	0.50–2.58	0.752	2.78	1.01–7.69	0.049
Yes	reference			reference		
Waist circumference (cm)						
<80	reference			reference		
≥80	2.22	1.23–4.00	0.008	1.65	0.78–3.47	0.189
Body mass index (kg/m^2^)						
<24	reference			reference		
≥24	2.51	1.35–4.66	0.004	2.74	1.23–6.12	0.014
Awareness of having HBP						
No	reference			reference		
Yes	3.40	1.10–10.53	0.034	5.06	1.39–18.49	0.014
Rural medical insurance						
No	reference			reference		
Yes	14.48	1.94–108.39	0.009	20.69	2.09–204.76	0.010

**Notes.**

CI confidence interval CVD cardiovascular disease HBP high blood pressure OR odds ratio

**Figure 2:**
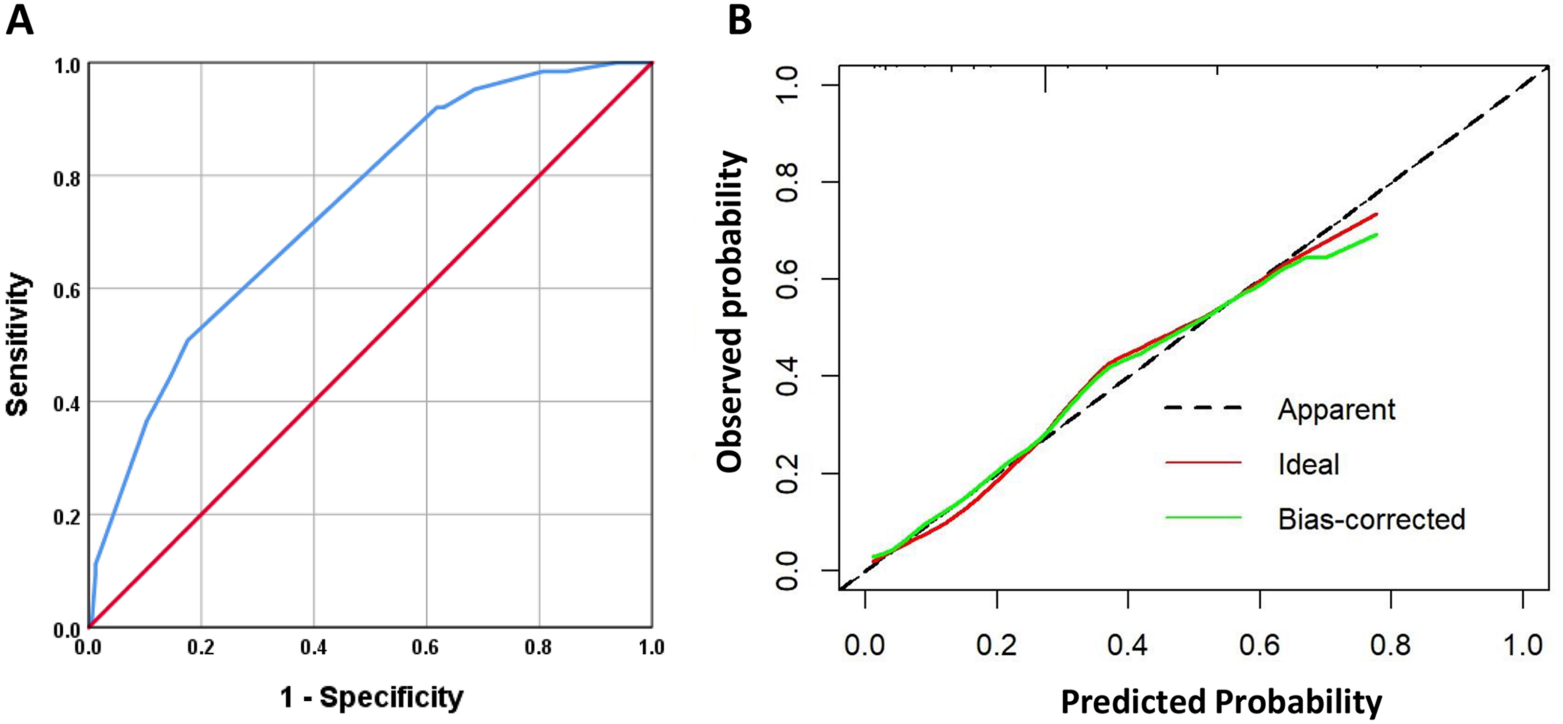
The receiver operating characteristic curve (ROC) (A) and calibration curve (B) of multivariable analysis model.

The multivariable logistic regression analysis (with all variables entered at the same time) showed that the significant factors associated with higher odds of HBP were having rural medical insurance (OR = 20.7, 95%CI [2.1–204.8]), physical activity less than three days per week (OR = 2.9, 95%CI [1.1–7.9]), being overweight or obese (OR = 2.7, 95%CI [1.2–6.1]), having parents without CVD or uncertain (OR = 2.8, 95%CI [1.0–7.7]), and awareness of having HBP (OR = 5.1, 95%CI [1.4–18.5]) (Table 4). No collinearity was observed among these included variables (all VIF <5). The five statistically significant variables above were incorporated into a logistic regression model. The AUC of this model was 0.742 (95%CI [0.673–0.811], Fig. 2A), and Fig. 2B presents the calibration curve. The Hosmer-Lemeshow test revealed *P* = 0.801.

Parental CVD history may directly increase the risk of HBP among migrant workers. A current awareness of having high blood pressure (being informed of HBP by a physician or nurse) may indirectly affect the prevalence of HBP by influencing physical activity, BMI, and purchase of rural medical insurance (Fig. S1).

## Discussion

This study investigated the prevalence, awareness, and factors associated with HBP (SBP ≥ 130 mmHg or DBP ≥ 80 mmHg) among young female migrant workers in Central South China. We found that approximately 27% of female migrant workers had HBP, and the majority of them were unaware of their condition. In total, 50% reported having good, very good, or excellent overall health status. Nearly one-third of the migrant women were overweight or obese, and 44% had central obesity. Many of these female migrants did not adhere to the recommended level of fruit or vegetable intake and physical activity. We also found that having rural medical insurance, physical activity less than three days per week, being overweight or obese, and being aware of having HBP were associated with the higher odds of HBP. The validation tests showed that the model’s results had good goodness of fit. The results have implications for the public health management of migrant women. The uncontrolled HBP among young Chinese female migrant workers suggested that this specific population needs health education promotion and specific programs.

In our study, the prevalence of HBP (27.2%) and hypertension (28.9%) according to the 2017 ACC/AHA guidelines were higher than both the recent 2017 prevalence report of 15.6% in Hunan province of Central South China (Wang et al., 2018) and the prevalence of 13.5% among Chinese women at reproductive age (Wang et al., 2017) based on the JNC-8 guidelines (SBP ≥ 140 mmHg or DBP ≥ 90 mmHg or use of antihypertensive medication) (James et al., 2014). Since an SBP of 130–139 mmHg or a DBP of 80–89 mmHg was included in the stage I diagnosis for hypertension according to the new 2017 ACC/AHA criteria, an additional 44 women (19.0% of the migrant workers) were classified as hypertensive. Therefore, the proportion of Chinese female migrant workers aged between 18 and 45 years classified as having hypertension would increase from 8.2% based on the JNC-8 guidelines to 27.2% based on the 2017 ACC/AHA guidelines, representing a remarkably higher prevalence of hypertension compared with the general population. Of note, comparison of prevalence studies should be interpreted with caution because of the differences in blood pressure thresholds over the years and across different guidelines from different countries.

Whether the new ACC/AHA standard is applicable for assessing the prevalence of hypertension in China is highly controversial. Several cardiovascular experts argued that the strategy of hypertension management must combine scientific evidence with the current socioeconomic capacity because of its high incidence and differences in each country or region based on blood pressure target achievement. Meanwhile, some researchers advocated for implementing early management of hypertension in China to address the increasing prevalence of hypertension in younger adults. Li et al. (2019) found that Chinese young adults (25–34 years) accounted for the highest proportion of newly diagnosed individuals with HBP (130–40 mmHg/80–90 mmHg according to the 2017 ACC/AHA hypertension guidelines). To reduce the national health burden of HBP-related diseases in the long term, the significantly high prevalence among young migrant female workers in our study should be considered a public health issue that should be further investigated.

Our results also showed that only a minority (11.1%) of the female migrant workers with current HBP were aware of their condition. The awareness level of currently or formerly having HBP in our subjects was far lower than among migrant workers reported by Li et al. (2017) (5.6% *vs.* 44.3%). This difference might be due to the general traits of the participants in the current study, who were younger and had lower levels of education (secondary school and above). Other studies showed that a disadvantaged socioeconomic status, including younger age, low level of education, having an agricultural or rural migrant occupation, and low income, were significantly associated with poor health literacy (Wang et al., 2015; Xie et al., 2019). Poor health literacy might influence an individual’s ability to understand hypertension and lead to disparities in access to hypertension care (Ahuja et al., 2018). Another explanation for this high unawareness of having HBP might be the disparity in utilization of public health resources because of economic or insurance limitations. A low socioeconomic status was more associated with HBP in women than men (Leng et al., 2015). In our study, most of the migrant female workers had a monthly income of ≤ USD 420, and nearly 14.2% had no insurance; however, 98.4% of those migrant women diagnosed with hypertension also had rural insurance. The rural forms of health insurance are distinct from those in the urban systems in China and are not transferable to the urban insurance system and/or may not be reimbursed in the same amount in the rural region. Thus, the possibility of willingness to receive medical treatment for these migrant workers in their new living environment is limited by their economic status and because their health insurance has higher rates of reimbursement for care received in rural and town hospitals and lower rates of reimbursement in city hospitals. Some migrants have to go back to rural areas for cheaper medical services. It is probably why having rural insurance had the largest OR for HBP. Theoretically, the direct effect of the medical insurance system is to reduce the medical burden of the insured so that they can get timely treatment for illnesses. Therefore, once people with medical insurance have health-related problems, they are less likely to be affected by economic factors. On the other hand, people without medical insurance tend not to seek medical treatment even if they know they have health conditions because their economic situation is not good enough to cope with the follow-up and treatment costs. But some migrant workers choose to buy medicines by themselves, considering the low cost of medical treatment and the low reimbursement rate of the whole, and the extra cost of transportation and loss of work when they return to the countryside. According to a Chinese survey, about 24.3% of migrant workers self-medicated, and 29.0% did nothing when they developed symptoms in the past month, which might be because the rural and urban insurance systems are not fully connected (Shao et al., 2018). Therefore, eliminating the regional disparities in rural and urban health insurance for female migrants and enhancing the health service availability will probably benefit the prevention and management of hypertension. But it also means increasing out-of-pocket costs for migrant workers.

Our study found that general obesity (determined by BMI) and a low level of physical activity but not central obesity (determined by WC) were independently associated with HBP. Epidemiological studies have demonstrated that obesity is one of the most important factors associated with hypertension (Nurdiantami et al., 2018; Susic & Varagic, 2017). Moreover, women with obesity are seven times more likely to develop hypertension than their normal-weight counterparts (Aucott et al., 2005). The high prevalence of obesity might result from limited adherence to the recommended dietary practices and physical activity levels. Rapid urbanization in China has contributed to lifestyle changes, including unhealthy dietary habits (intake of more refined foods and sugary drinks) and reduced physical activity, which have led to the increased prevalence of general and central obesity and the development of hypertension (Schwingshackl et al., 2017; Su, Sun & Xu, 2018; Yan et al., 2016). Observational data indicate that maintaining a minimum of 150 min of moderate physical activity per week for adults aged ≥ 18 years would potentially prevent 46% of inactivity-related deaths (Mok et al., 2019). The proportion of female migrant workers who met the recommended level of physical activity was only 18.5%, which was lower than the average national rate of 22.8% (Tian et al., 2016). According to a 20-year cohort study in China, physical activity among adults has decreased by half from 1991 to 2011, with much steeper declines in women than in men. Each new generation had lower activity levels than the previous one (Zang & Ng, 2016). Strong evidence has demonstrated that physical activity could reduce blood pressure among adults with prehypertension and hypertension (Pescatello et al., 2019). Additionally, physical inactivity independently contributes to the development of CVD in women (Li et al., 2006). These findings highlight the importance of developing a healthy lifestyle program to increase awareness and reduce obesity and hypertension among female migrant workers.

Many studies explored the increases in hypertension prevalence by age (Bundy & He, 2016; Gao et al., 2013; Hu, Gao & Liu, 2019; Zhou, 2002). The prevalence of HBP has been recently reported to be high in young Chinese people. The prevalence of hypertension in younger adults (20–44 years) doubled from 2002 to 2010 (12% *vs.* 23% in men, 6% *vs.* 14% in women), which was much greater than the growth rate in older adults (29% *vs.* 46% in aged 45 years or above) (Bundy & He, 2016). The mean age of Chinese migrant workers was 39 years, and 53.9% of them were aged 40 and below. Furthermore, women accounted for 34.5% of Chinese migrant workers (Department of Service and Management of Migrant Population NHaFPCoC, 2017). The study by Guan in 2009 showed that about 25.0% of Chinese migrants aged at least 18 years had prehypertension (19.9%) or hypertension (5.1%) (Guan, 2018). In addition, the prevalence of hypertension rapidly increased to 16.3% in 2012 among Chinese migrant workers aged 18–59 years (Bi et al., 2016). Li et al. (2017) found that in 2015, the prevalence of hypertension was similar between migrants and urban residents (17.3% *vs.* 18.1%). Although many female migrant workers are more likely to be mainly engaged in manual labor, have a low level of education, and lack access to healthcare services (Zheng et al., 2018), the studies on key health issues, including hypertension and CVD, are limited in this population (Fang et al., 2017; Yang et al., 2015b; Zhang et al., 2018). We also found that age ≥ 35 years was associated with HBP in the bivariable analysis but not in the multivariable one. Jin et al. reported that the prevalence of HTN in the migrant population aged <35 years was lower than that in the older population aged ≥ 35 years (5.2% *vs.* 28.4%) in Hunan in 2012 (Donghui et al., 2015). Similar conclusions were also reported in other studies in China (Liu et al., 2017; Zhang et al., 2013). With increasing age, the stiffness of the aorta and arterial walls increases, which contributes to the high prevalence of hypertension in the older group (Sabbahi et al., 2016; Yoo et al., 2018). Although aging cannot be reversed, increasing the awareness of hypertension and promoting a healthy lifestyle among female migrant workers may be an effective way to delay the development of this condition, especially for those aged older than 35 years.

The bivariable analysis suggested that the difference was not significant in terms of the odds of HBP in female migrant workers whose parents did not have (or were uncertain of having) a history of CVD and those whose parents had CVD, but the association was significant in the multivariable analysis. Indeed, increased awareness of family history might increase an individual’s awareness of the disease. Future studies should explore other potential mechanisms for hypertension among this population. Of note, it is common in medical research to include in a multivariable analysis only the variables that were significant in the bivariable analyses. Still, this practice can be misleading because some of the association of specific variables with the outcome might become significant only when considering the other variables. Indeed, such a circumstance can occur in the presence of groups with different sizes, missing data, an extreme within-group variation, and the presence of interaction (Lo et al., 1995). Although the present study was not designed to determine causality, this association was significant in the multivariable analysis but not in the bivariable one. Findings regarding this association are hypothesis-generating and should be explored in future studies.

Although being aware of having HBP, blood pressure control was unsatisfactory among young female migrants, with a control rate of 46.2% (6/13). Public health workers should pay more attention to young female migrants, educate them about the increasing prevalence of HBP and the optimal goal for blood pressure control, and implement measures to reduce their risk factors for active prevention of hypertension. Improving knowledge and promoting a healthy lifestyle are crucial to alleviating the growing hypertension burden in the female migrant population. There were many female workers with obesity and HBP. This phenomenon reveals that prevention strategies are still needed for female workers. The awareness of whether one and their parents have HBP needs to be improved, which has a certain relationship with the health education level of female workers. It is necessary to strengthen health literacy interventions related to the prevention and treatment of chronic diseases.

### Limitations

This is one of the first studies to examine the prevalence, awareness, and factors associated with HBP in young female migrant workers in Central South China. However, the study also has some limitations. First, a causal relationship could not be established due to the cross-sectional design. Second, our study included only female migrant workers aged between 18 and 45 years, thus limiting the generalizability of our results to other populations. In addition, the present study did not conduct surveys on female workers in every category of occupation. Still, it adopted the principle of convenient sampling and volunteers from large, medium, and small enterprises with ≥ 50% of women, which certainly introduced selection biases. Third, family and personal health history were based on self-reports, introducing recall biases. HBP medications were also based on the participant’s response (yes or no) to a question, without asking the types of medications or confirming using the participants’ pill bottles from home. Fourth, for accurate measurement of blood pressure, the 2017 AHA guideline suggested using an average of ≥ 2 readings obtained on ≥ 2 occasions to estimate the individual’s level of BP (Whelton et al., 2018). The failure to measure blood pressure on another occasion was a limitation in our study. Fifth, this study was not designed to take into account white coat HBP (which could overestimate the prevalence of HBP) or masked hypertension (which could underestimate the prevalence of HBP). Finally, and most importantly, the patients were divided based on having HBP or not. Therefore, some patients who self-reported hypertension history with controlled blood pressure were grouped as non-HBP. Nevertheless, the results indicated that only 6% of the hypertensive patients had controlled BP.

## Conclusions and Implications for Practice

This study revealed a high prevalence of HBP among young Chinese female migrant workers, as well as a high frequency of being unaware of their condition and some associated factors (rural medical insurance, awareness of having HBP, physical inactivity, and overweight/obesity). The uncontrolled HBP among young Chinese female migrant workers suggested that health education needs further promotion in such a population.

## Supplemental Information

10.7717/peerj.13365/supp-1 Supplemental Information 1 Directed acyclic graph for the illustration of possible causal relationship

10.7717/peerj.13365/supp-2 Supplemental Information 2 Raw measurements

## Abbreviations

CVD: cardiovascular disease
JNC-8: Eighth Joint National Committee
SBP: systolic blood pressure
DBP: diastolic blood pressure
HBP: having high blood pressure
ACC: American College of Cardiology
AHA: American Heart Association
CI: confidence interval
WC: waist circumference
HTN: hypertension
SDs: standard deviations
OR: odds ratio

## Additional Information and Declarations

### Data Availability

The following information was supplied regarding data availability: The raw measurements are available in the Supplementary File.
