# Supplementary figures and images for: Prevalence, awareness, and associated factors of high blood pressure among female migrant workers in Central South China
Source: PeerJ. 2022 May 4;10:e13365. doi: 10.7717/peerj.13365 (PMC9078134; doi:10.7717/peerj.13365)

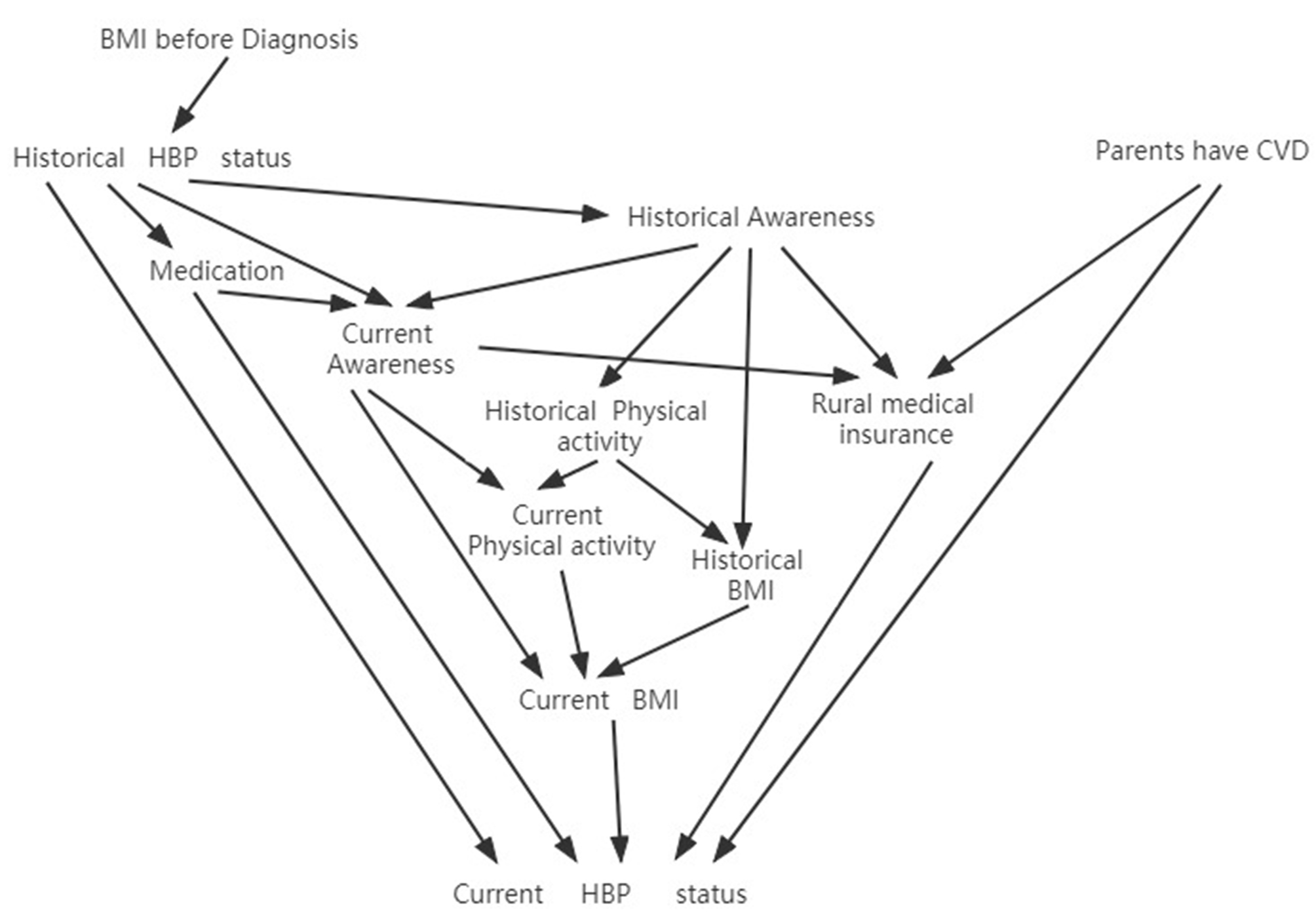

Supplement: Supplemental Information 1 [file peerj-10-13365-s001.png]
